# Multi-Habitat Based Radiomics for the Prediction of Treatment Response to Concurrent Chemotherapy and Radiation Therapy in Locally Advanced Cervical Cancer

**DOI:** 10.3389/fonc.2020.00563

**Published:** 2020-05-05

**Authors:** Mengjie Fang, Yangyang Kan, Di Dong, Tao Yu, Nannan Zhao, Wenyan Jiang, Lianzhen Zhong, Chaoen Hu, Yahong Luo, Jie Tian

**Affiliations:** ^1^School of Artificial Intelligence, University of Chinese Academy of Sciences, Beijing, China; ^2^CAS Key Laboratory of Molecular Imaging, Institute of Automation, Chinese Academy of Sciences, Beijing, China; ^3^Cancer Hospital of China Medical University, Shenyang, China; ^4^Liaoning Cancer Hospital & Institute, Shenyang, China; ^5^Beijing Advanced Innovation Center for Big Data-Based Precision Medicine, School of Medicine, Beihang University, Beijing, China

**Keywords:** cervical cancer, MRI, radiomics, treatment response prediction, concurrent chemotherapy and radiation therapy, precision medicine

## Abstract

**Objectives:** To develop a radiomic model based on multiparametric magnetic resonance imaging (MRI) for predicting treatment response prior to commencing concurrent chemotherapy and radiation therapy (CCRT) for locally advanced cervical cancer.

**Materials and methods:** The retrospective study enrolled 120 patients (allocated to a training or a test set) with locally advanced cervical cancer who underwent CCRT between December 2014 and June 2017. All patients enrolled underwent MRI with nine sequences before treatment and again at the end of the fourth week of treatment. Responses were evaluated by MRI according to RECIST standards, and patients were divided into a responder group or non-responder group. For every MRI sequence, a total of 114 radiomic features were extracted from the outlined tumor habitat. On the training set, the least absolute shrinkage and selection operator method was used to select key features and to construct nine habitat signatures. Then, three kinds of machine learning models were compared and applied to integrate these predictive signatures and the clinical characteristics into a radiomic model. The discrimination ability, reliability, and calibration of our radiomic model were evaluated.

**Results:** The radiomic model, which consisted of three habitat signatures from sagittal T2 image, axial T1 enhanced-MRI image, and ADC image, respectively, has shown good predictive performance, with area under the curve of 0.820 (95% CI: 0.713–0.927) in the training set and 0.798 (95% CI: 0.678–0.917) in the test set. Meanwhile, the model proved to perform better than each single signature or clinical characteristic.

**Conclusions:** A radiomic model employing features from multiple tumor habitats held the ability for predicting treatment response in patients with locally advanced cervical cancer before commencing CCRT. These results illustrated a potential new tool for improving medical decision-making and therapeutic strategies.

## Introduction

Cervical cancer is one of the leading causes of cancer-related mortality in women, among which locally advanced cervical cancer has poor prognosis (1, 2). According to National Comprehensive Cancer Network (NCCN) guidelines, concurrent chemotherapy and radiation therapy (CCRT) with cisplatin-based chemotherapy is a primary treatment for patients with locally advanced cervical cancer (3); the 5-years survival rate can reach 60–80% (4). However, if first-line CCRT fails, the extended CCRT treatment period inevitably delays the commencement of other potentially effective treatments (5). In addition, CCRT has many side effects. Extra-pelvic irradiation affects the bones that contain more than half the body's proliferative active bone marrow volume and may cause myelosuppression. Platinum-based CCRT can aggravate myelosuppression, but treatment delay or interruption affects its efficacy (6). Therefore, the prediction of response to CCRT before treatment commences may help us to decide whether to choose CCRT as first-line treatment. Moreover, response prediction can guide personalized medicine by selecting patients who are most sensitive to CCRT.

In clinical practice, assessing response to CCRT is often based on tumor biopsy histology during or after treatment, which provides no value in predicting the outcome for a CCRT decision. Magnetic resonance imaging (MRI) is routinely used for non-invasive diagnosis, treatment planning, and assessing treatment response of locally advanced cervical cancer (7). There are numerous MRI sequences, such as T1 and T2, dynamic contrast enhanced MRI, and diffusion-weighted MRI (DWI). Although these MRI sequences could assess the likely response based on morphological changes after treatment (8), this is still a lagging indicator (9). Pretreatment methods for predicting the response of CCRT are still lacking.

Radiomics is an emerging field that aims to extract large amounts of quantitative information from medical images that may not be easily obtained or quantified by traditional means (10–13). This kind of technique is able to capture features that reflect intratumoral heterogeneity, which is thought to be related to sensitivity to chemotherapy, radiotherapy, and other treatments (14). Therefore, it has been widely used to predict treatment response in nasopharyngeal carcinoma (15), rectal carcinoma (16), and lung cancer (17).

**Figure 1:**
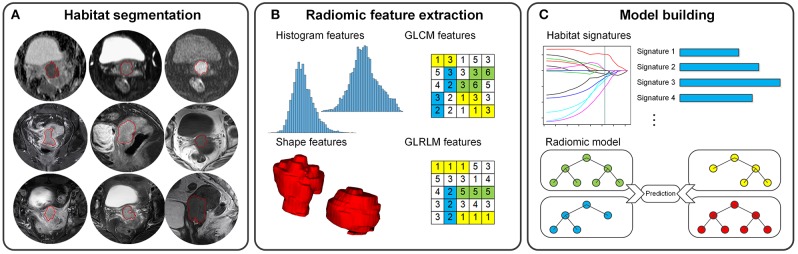
Radiomics pipeline for the prediction of CCRT response in locally advanced cervical cancer. **(A)** Nine tumor habitat segmentations. **(B)** 3D radiomic feature extraction. **(C)** Development of habitat signatures and radiomic model.

In this study, we aimed to develop and validate a radiomic model for predicting treatment response to CCRT in locally advanced cervical cancer using pre-treatment MRI data (Figure 1).

## Materials and Methods

Ethical approval for this retrospective study was granted by the ethics committee of the Cancer Hospital of China Medical University, and the informed consent for patients was waived.

### Patients

One hundred and forty consecutive patients with cervical carcinoma, treated between December 2014 and June 2017, were enrolled according to the following inclusion criteria: (1) histologically proven (biopsy) cervical carcinoma and clinical diagnosis of locally advanced cervical cancer according to the International Federation of Gynecology and Obstetrics (FIGO) classification (FIGO stage IB2-IIB) (18); (2) no medical treatment or CCRT prior to the pre-treatment MRI; (3) no other malignancies during the study; (4) CCRT administered based on the NCCN Clinical Practice Guidelines in Oncology-Cervical Cancer Guideline) (3); and (5) MRI performed again at the end of the fourth week of treatment for response evaluation. 20 patients were excluded from the study according to the exclusion criteria: (1) missing clinical data (i.e., the clinical characteristics listed in Table 1; *n* = 5); (2) image artifacts (*n* = 3); (3) >3 slices of tumor volume (the slice thickness = 4 mm; *n* = 4); and (4) more than 1 week between performing the pre-treatment MRI and commencing CCRT (*n* = 8). Therefore, a total of 120 patients met these criteria. Computer random number generation was used to split half of patients into the training set [*n* = 60, mean age, 51.5 ± 9.2 (range, 27–76) years], and the rest patients into the test set [*n* = 60, mean age, 53.7 ± 9.0 (range, 34–75) years].

**Table 1:** Clinical characteristics of patients in the training and test sets.

**Characteristics**	**Training set**		***P***	**Test set**		***P***
	**Responder group**	**Non-responder group**		**Responder group**	**Non-responder group**	
Age (Mean ± SD)	50.6 ± 9.1	52.6 ± 9.3	0.401	52.1 ± 8.9	56.1 ± 8.9	0.098
Pregnancy Num (Mean ± SD)	3.4 ± 1.6	3.0 ± 2.0	0.228	2.9 ± 1.6	3.0 ± 1.0	0.578
Parturition Num (Mean ± SD)	1.3 ± 0.7	1.4 ± 1.0	0.250	1.5 ± 0.7	1.5 ± 0.7	0.703
Abortion Num (Mean ± SD)	2.1 ± 1.6	1.5 ± 1.7	0.053	1.5 ± 1.6	1.4 ± 1.1	0.930
First age of sexual intercourse (Mean ± SD)	23.0 ± 2.7	23.4 ± 2.7	0.641	22.4 ± 4.6	23.5 ± 3.3	0.461
Family history of cancer, n (%)			0.506			0.634
YES	2(5.7%)	0(0.0%)		4(11.1%)	1(4.2%)	
NO	33(94.3%)	25(100.0%)		32(88.9%)	23(95.8%)	

*n, number; P-value was derived from the univariate association analysis between each characteristic and responses*.

All patients underwent standard CCRT according to NCCN guidelines (3). The detailed treatment schemes are shown in Supplementary Material.

### MRI Protocols

All patients underwent the pre-treatment multiparametric MRI examinations before CCRT (within 1 week) and had a follow-up MRI. The MRI images were acquired using a 3.0T MR system (Siemens Magnetom Verio, Erlangen, Germany). The scanning range was set to cover the whole pelvis, and the scanning positioning line was consistent. The detailed MRI scanning protocols are shown in Supplementary Material and Table S1. Patients were scanned with sagittal T2, axial T1, axial T2-FS, DWI with b = 0, and b = 800, apparent diffusion coefficient (ADC), and enhanced-MRI (sagittal, axial, and coronal directions). The follow-up MRI scans used the same parameters with the pre-treatment MRI. In this study, we retrieved the pre-treatment MRI images from a picture archiving and communication system (PACS; Neusoft, Shenyang, China) for segmentation and analysis.

### Evaluation of Treatment Outcome

Referring to Response Evaluation Criteria in Solid Tumors standards, two highly experienced radiologists in gynecological MRI jointly analyzed treatment response (19). Responses can be classified into groups of CR (Complete response, absence of residual tumor), PR (partial response, the longest diameter of the tumor was <70% of the original size), PD (progressive disease, at least a 20% increase in the longest tumor diameter compared to the original size), and SD (stable disease, neither sufficient shrinkage for PR nor sufficient increase for PD). Patients were thus divided into a responder group, including CR and PR, or a non-responder group, including PD and SD in this study. The pre-treatment MRI before CCRT was used to predict responder or non-responder in this study.

### Tumor Habitat Segmentation and Feature Extraction

Habitats are the tumor regions/subregions in different image sequences (11). In this study, the highlighted tumor volume in one MRI sequence was defined as a tumor habitat. Three-dimensional (3D) regions of interest (ROI) of tumor habitat was segmented manually by a radiologist with 10 years' experience (radiologist 1) using ITK-SNAP software (version 3.6.0; http://www.itk-snap.org). In details, the ROI were drawn on each of the 2D slices to include the entire tumor volume. Nine tumor habitats were delineated on the nine pre-treatment MRI sequences. In addition, we randomly chose 25 patients from the training set and asked another radiologist (radiologist 2) with 15 years' experience to segment habitats for analyzing the reproducibility of features.

We normalized image intensity and calculated quantitative radiomic features from these habitats. The radiomic features contained 114 3D descriptors for each habitat, including shape features, histogram features, gray-level co-occurrence matrix (GLCM) features, and gray-level run-length matrix (GLRLM) features. Referring to the Image Biomarker Standardization Initiative (IBSI) (20), the features were extracted using an in-house developed toolbox performed in MATLAB (version 2017a; MathWorks, Natick, MA, USA). The details of radiomic feature extraction are described in Supplementary Material.

### Habitat Signature and Radiomic Model Building

Based on the training set, we built nine habitat signatures and a radiomic model in this study. For each habitat, a three-step feature selection procedure was used to remove the non-reproducible or redundant features and find the most predictive ones in the training set. First, we analyzed the stability of radiomic features from the re-segmentation data using inter-class correlation coefficient (ICC). Features with ICCs <0.8 were removed. Second, Pearson correlation coefficients between every pair of features were calculated to measure the linear correlations. To reduce redundancy, for the pairs that yielded correlation coefficients > 0.6, we only retained the more stable one (i.e., with high ICC). Then, the least absolute shrinkage and selection operator (LASSO) logistic regression model (21) was utilized to assess the importance of features and obtain the most predictive combination as the habitat signature. Therefore, we obtained nine habitat signatures from the nine MRI sequences.

To build the radiomic model, we compared three kinds of machine learning models, including support vector machine, random forest, and logistic regression model, with the habitat signatures and clinical characteristics as input variables. To select the best model as well as the optimized hype-parameters, and to remove the meaningless or redundant variables, multiple three-fold cross-validation with the grid search was implemented on the training set. The average accuracy was used as the evaluation criteria. We then obtained the final radiomic model by re-training the selected model with the whole training set.

### Statistical Analysis and Performance Evaluation

Clinical characteristics with potential prognostic outcomes were identified in univariate analysis on the basis of the chi-square test or Fisher's exact test for categorical variables and the *t*-test or Mann-Whitney *U* test for continuous variables. Multivariable logistic regression analysis was implemented to identify the independent predictors. The statistical significance used *P* < 0.05.

The quantitative discrimination performance was assessed using the receiver operator characteristics (ROC) analysis. The area under the curve (AUC) with 95% confidence interval (95% CI) was calculated. The point on the ROC curve of the training set yielding the highest Youden's index was selected as the cut-off value. Then, the accuracy of the model was assessed. We implemented calibration curve analysis with the Hosmer-Lemeshow test to measure the agreement between the predicted probability of treatment response and the observed results. The consistency test for the radiomic model was conducted by comparing its outputs based on the segmentation of the two radiologists (i.e., the re-segmentation data). Furthermore, to fully investigate the performance and robustness of the proposed multi-habitat based radiomics, we randomly split the 120 patients into training sets or test sets 20 times. Then, the model was built and validated repeatedly. The methods of statistical analysis were conducted using the R software (version 3.5.0; http://www.rproject.org).

## Results

### Clinical Characteristics

The clinical characteristics of patients are summarized in Table 1. There were no significant differences between the responder and non-responder groups in the training or test sets in terms of any of the clinical characteristics, based on univariate analysis (*P* > 0.05).

### Feature Selection and Modeling

Among the 114 original radiomic features normalized by z-score transformation in each habitat, key features were obtained after the three-step feature selection methodology (Table 2). The selected features captured the intensity, shape, and texture pattern of the tumors. Then, nine habitat signatures were built via LASSO logistic regression, respectively. All the signatures showed significant associations with the treatment response, yielding AUCs ranging from 0.611 to 0.741 in the training set. Beginning with the habitat signatures and the clinical characteristics, grid search was implemented on three machine learning models, respectively. The random forest combining the habitat signatures from sagittal T2 image, axial T1 enhanced-MRI image, and ADC image yielded the highest average accuracy in multiple cross-validations, and therefore was selected to construct the radiomic model. The radiomic scores, which represented the predicted possibility of response to CCRT, were calculated via radiomic model for all the patients.

**Table 2:** The remaining features after a three-step feature selection methodology.

**Habitats**	**Number of features after step 1**	**Number of features after step 2**	**Selected features in habitat signature**
Sagittal T2	96	11	X_GLRLM_LRHGLE
Axial T1	93	9	X_GLRLM_RP
Axial T2-FS	97	11	XLL_GLRLM_SRLGLE
Axial DWI b=0	102	14	X_GLRLM_LRHGLE
Axial DWI b=800	99	13	X_GLRLM_LRE, X_GLRLM_SRLGLE
ADC	95	10	X_GLCM_variance
Sagittal T1 enhanced-MRI	97	8	XLL_GLRLM_RLN
Axial T1 enhanced-MRI	103	9	XHH_GLRLM_RP, Surface_area, XLL_H_skewness, X_GLCM_dissimilarity
Coronal T1 enhanced-MRI	73	10	X_GLCM_homogeneity2

*X, original image; XLL, original image filtered directionally with low-pass filter along x and y directions; XHH, original image filtered directionally with high-pass filter along x and y directions; H, histogram; LRHGLE, long run high gray level emphasis; RP, run percentage; SRLGLE, short-run low-gray level emphasis; LRHGLE, long-run high-gray level emphasis; LRE, long-run emphasis; RLN, run length non-uniformity*.

### Performance Evaluation of Radiomic Model

**Figure 2:**
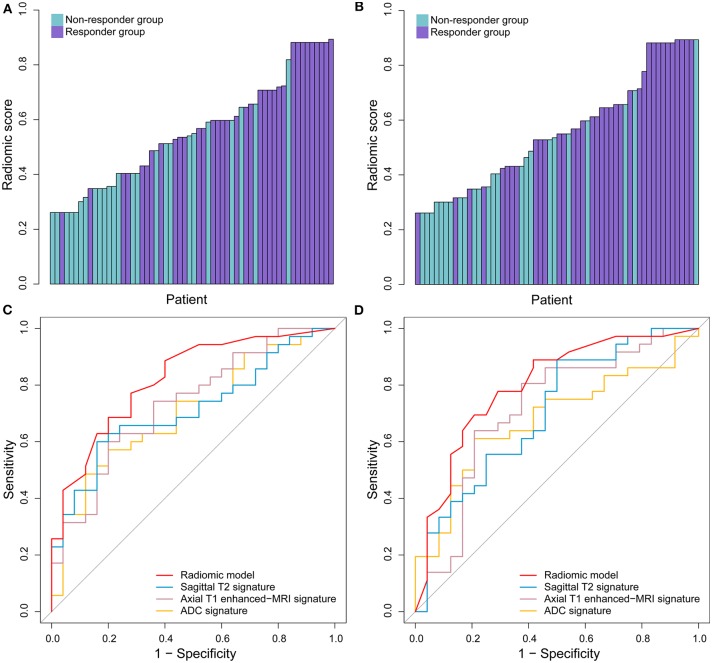
The performance of radiomic model in predicting the response to treatment. The radiomic model scores in the training set **(A)** and test set. **(B)** The ROC curves of radiomic model and selected three single signatures in the training set **(C)** and test set **(D)**.

**Figure 3:**
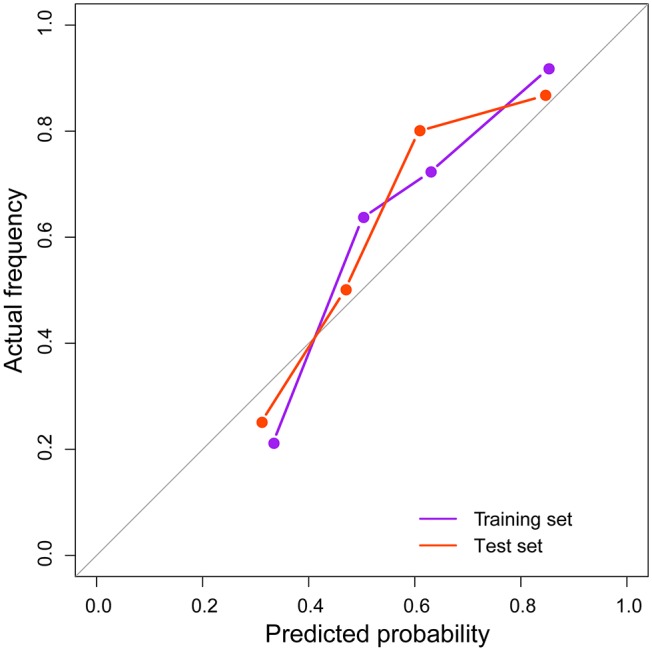
Calibration curves of the radiomic model in the training and test sets.

In the ROC analysis, the radiomic model yielded satisfactory prediction performance in the training set (AUC = 0.820, 95% CI: 0.713–0.927) and test set (AUC = 0.798, 95% CI: 0.678–0.917). It had better predictive performance than any single habitat signature in both training and test sets, which are listed in Table 3 in detail. The radiomic model was the only factor that reached statistical significance for predicting treatment response in the multivariable analysis including the clinical characteristics. The distribution of radiomic model score and the treatment response for each patient, as well as the ROC curves, are shown in Figure 2. With the cut-off value of 0.520, the classification accuracies were 0.750 in both the training and test sets. The calibration curves of the radiomic model showed good agreement between the predicted and actual probabilities of treatment response in the two sets (Figure 3). The Hosmer-Lemeshow test suggests that there is no significant departure (*P* = 0.368 and 0.437, respectively). The radiomic scores of the re-segmentation data demonstrated that there was substantial inter-radiologist agreement for the model (ICC = 0.809). Meanwhile, the model's performances were relatively consistent on the segmentations of the two radiologists (AUCs = 0.903 and 0.803, respectively; Delong test *P* = 0.116). Moreover, we repeatedly randomly split the whole data into paired training and test sets (*n* = 60 in both sets), and used each of the training/test sets to completely rebuild and evaluate a model. In 20 cross-validation experiments, the AUCs on the holdout test set ranged from 0.776 to 0.849 with a mean value of 0.804.

**Table 3:** AUCs of the habitat signatures and radiomic model in the training and test sets.

**Models**	**Training set**	**Test set**
Sagittal T2 signature	0.713	0.704
Axial T1 signature	0.653	0.631
Axial T2-FS signature	0.680	0.683
Axial DWI b=0 signature	0.675	0.594
Axial DWI b=800 signature	0.741	0.676
ADC signature	0.704	0.678
Sagittal T1 enhanced-MRI signature	0.611	0.650
Axial T1 enhanced-MRI signature	0.734	0.713
Coronal T1 enhanced-MRI signature	0.679	0.567
Radiomic model	0.820	0.798

## Discussion

In this study, a MRI radiomic model was built based on multi-habitat signatures to predict response to CCRT in locally advanced cervical cancer. The radiomic model, which consisted of three habitat signatures, had good predictive performance in both training and test sets. Moreover, our model performed better than those with single habitat signature or clinical characteristics.

We used multi-habitat signatures from different MRI sequences. These sequences reflected different characteristics of tumors, including morphological, physiological, and metabolic characteristics. DWI MRI sequence uses ADC for quantification and comparison in functional imaging (22), which may reflect the biological heterogeneity of tumors. DWI-ADC changes early during radiotherapy for cervical cancer, predicting tumor responses on MRI (23). The signatures from DWI scans apparently conflict with the observations on routine MRI. This could be the result of DWI scans being assessed only qualitatively (e.g., signal intensity, texture), as there is no established ADC value that is a diagnostic for lesion tissue. T2 MRI images can detect tumor intensity, and T1 enhanced-MRI images may reflect intratumoral heterogeneity (15).

Intratumor heterogeneity was thought to influence the CCRT response (14, 24). Most locally advanced cervical tumors present with high intratumoral heterogeneity in virtually all distinguishable phenotypes, such as proliferation, vascularity, metabolism, oxygenation, etc., which directly suggests tumor resistance to therapy (25). Although histopathological samples can be acquired by surgery or biopsy, tumor heterogeneity may lead to sampling errors. Such data are available only after completion of all pre-operative treatment and surgery and cannot be used as guidance for adjusting the therapeutic approach before or during treatment. Identification of a biomarker that could illuminate the individual's biological behavior and aid in identifying those who are less likely to benefit from CCRT and should thus undergo alternative treatment or intensive follow-up regimens would help with further classifications of locally advanced cervical cancers. Predicting tumor response to CCRT accurately before treatment has been a major challenge in the era of personalized medicine, and no generally accepted biomarker for cervical cancer has been reported (26). Being a non-invasive and low-cost method, radiomics may offer an innovative solution to this problem.

In this study, we explored 1,026 quantitative features to uncover the tumor characteristics (27, 28). A main challenge of this study is to reduce high dimensional feature set to a representative subset that is most closely associated with the responses. For each habitat, we reduced the 114 radiomic features to a signature with a few key features. Multiple cross-validation with three machine learning methods was then applied to the nine signatures and three signatures were selected for model building. These steps could reduce the complexity of the model and avoid overfitting.

The incidence of cervical carcinoma is related to certain risk factors, such as early sexual intercourse, frequent pregnancies, abortion, births, and a family history of cancer (29). We included these factors into the model, but they proved to be less significant (*P* > 0.05).

This study had some limitations. First of all, our study may carry some degree of selection bias. All data were obtained from a single research center, and patients may not have been randomized strictly. Further validation of the method based on external centers and large-scale cohorts is needed. Secondly, the selection of patients in the advanced clinical stage may limit the generalizability of our findings to other stages. Additionally, to ensure the reproducibility and stability of the radiomic model, the same scanning sequences and parameters were used for all patients. Therefore, the model needs to be tested on data with different MRI machines and scanning parameters.

In summary, we have identified an association between the habitat signatures and responses to CCRT in locally advanced cervical cancer. This association may have a clinical impact on selecting individualized therapies for patients with a rather reliable predicted outcome, before or during treatment.

## Data Availability Statement

The datasets for this article are not publicly available because of patient information privacy. Requests to access the datasets should be directed to JT, jie.tian@ia.ac.cn.

## Ethics Statement

Ethical approval for this retrospective study was granted by the ethics committee of the Cancer Hospital of China Medical University, and the informed consent for patients was waived.

## Author Contributions

MF, YK, and DD contributed equally to this work. JT and YL conceived and launched this study. MF, DD, and CH designed the medical and statistical analysis. YK, TY, NZ, and WJ collected cases and implemented the control of image quality and clinical diagnosis. MF and DD analyzed the data and carried out statistical experiments. MF, YK, DD, and LZ provided result interpretation. MF, YK, and DD wrote the first draft of this manuscript. LZ, JT, and YL revised and edited the final version. All authors reviewed and approved the manuscript.

## Conflict of Interest

The authors declare that the research was conducted in the absence of any commercial or financial relationships that could be construed as a potential conflict of interest.

## Abbreviations

ADC: apparent diffusion coefficient
AIC: Akaike's information criterion
AUC: area under the curve
CCRT: concurrent chemotherapy and radiation therapy
CI: confidence interval
CR: complete response
DWI: diffusion-weighted MRI
FIGO: International Federation of Gynecology and Obstetrics
GLCM: gray-level co-occurrence matrix
GLRLM: gray-level run-length matrix
IBSI: Image Biomarker Standardization Initiative
ICC: inter-class correlation coefficient
LASSO: least absolute shrinkage and selection operator
MRI: magnetic resonance imaging
NCCN: National Comprehensive Cancer Network
PD: progressive disease
PR: partial response
ROC: receiver operator characteristics
SD: stable disease
VIF: variance inflation factor.
