# Supplementary material for: Multi-Habitat Based Radiomics for the Prediction of Treatment Response to Concurrent Chemotherapy and Radiation Therapy in Locally Advanced Cervical Cancer
Source: Front Oncol. 2020 May 5;10:563. doi: 10.3389/fonc.2020.00563 (PMC7214615; doi:10.3389/fonc.2020.00563)
Supplement: Supplementary file 1 [file Table_1.DOCX]

Supplementary Material

# CCRT scheme

All patients underwent standard radiotherapy in combination with concurrent cisplatin-based chemotherapy, according to NCCN guidelines. All patients received chemotherapy (cisplatin or cisplatin plus paclitaxel/docetaxel), as follows. 1) Four cycles of bi-weekly cisplatin (40 mg/m2); 2) cisplatin (60-80 mg/m2) plus paclitaxel (135-175 mg/m2), d1; 1 cycle of 21 days, repeated this 21-28 days; 3) docetaxel (135-175 mg/m2), d1; cisplatin (60-80 mg/m2), d2; 1 cycle of 21 days, repeated this 21-28 days. For radiotherapy, external beam radiation therapy (EBRT) was used. EBRT was delivered at a total dose of 45-50 Gy, with a daily dose of 1.8-2.0 Gy, 5 days per week. Depending on condition, patients with pelvic lymph node metastasis received correspondingly more radiation.

# MRI protocols

Before MRI examinations, the patients drank sufficient water to fill the bladder moderately. All the MR images were obtained on a clinical whole-body 3.0-T MR scanner (Siemens Magnetom Verio, Erlangen, Germany) with a phased-array 8-channel sensitivity-encoding abdominal coil. Patients were placed in a supine position, head-first, and used breathing control. The scan covered the whole pelvis. Patients were scanned with sagittal T2, axial T1, axial T2-FS, DWI with b=0, and b=800, Apparent Diffusion Coefficient (ADC), and enhanced-MRI (in the sagittal, axial, and coronal directions). The entire tumour volume was within the field of view.

MRI scanning sequences and parameters were in Table S1.

# Radiomic feature extraction

Two-step image normalization was implemented before feature extraction: bicubic resampling was used to standardize the image scale, resulting in a pixel size of 0.5 mm × 0.5 mm; the intensity value transformation (scaling and shifting) was used to minimize the discrepancy of intensity distributions among patients.

A filtering process was performed to implement image smoothing and image difference. Separable filtering was used to avoid the multi-dimensional convolution. The convolution was performed with a low-/high-pass “Coiflet 1” wavelet filter along x-/y-direction separately. Consider L and H to be a low-pass and high-pass functions respectively, X to be the original image and the filtered results of X to be labelled as XLL and XHH. Two new images were obtained by filtering the original image.

In this study, a total of 114 candidate radiomic features were generated from the tumour habitat in each MRI sequence. Firstly, 3 shape features were calculated based on the segmentation. Then, 14 histogram features, 12 gray-level co-occurrence matrix (GLCM) features and 11 gray-level run-length matrix (GLRLM) features were generated from the image without/after filtration. Therefore, for each tumour habitat, a total of 114 (3+3×[14+12+11]) radiomic features were extracted. The features used in our study were referenced from [1-2].

**Appendix Tables**

**Table S1.** MRI sequences and parameters

| Parameters | Sequences | | | | | | |
| --- | --- | --- | --- | --- | --- | --- | --- |
|  | Sagittal T2 | Axial T1 | Axial T2-FS | DWI (b=0,800) | Contrast-enhanced sagittal T1 | Contrast-enhanced axial T1 | Contrast-enhanced coronal T1 |
| TR(ms) | 3800 | 550 | 550 | 3500 | 3.1 | 3.23 | 3.2 |
| TE(ms) | 26 | 13 | 3 | 93 | 1.25 | 1.22 | 1.23 |
| Slice Thickness(mm) | 4 | 4 | 4 | 4 | 4 | 4 | 4 |
| Acquisition Matrix | 320×320 | 320×320 | 320×320 | 320×256 | 202×384 | 195×320 | 202×384 |
| FOV(mm) | 448×396 | 400×400 | 400×400 | 400×400 | 400×400 | 400×400 | 400×400 |
| Inter Slice Gap | 1 | 1 | 1 | 1 |  |  |  |
| NSA | 2 | 2 | 2 | 2 | 2 | 2 | 2 |
| Contrast Agent |  |  |  |  | Gd-DTPA | Gd-DTPA | Gd-DTPA |

*Note*: FOV, field of view; NSA, number of signals averaged; Gd-DTPA, gadolinium diethylenetriaminepentaacetate acid (administered as a fast bolus injection in a total dose of 0.1 mmol/kg bodyweight, at a rate of 3ml/s, followed by a saline solution flush of 20ml)

**References**

[1] Aerts HJ, Velazquez ER, Leijenaar RT, Parmar C, Grossmann P, Carvalho S, et al. Decoding tumour phenotype by noninvasive imaging using a quantitative radiomics approach. Nature communications. 2014;5:4006.

[2] Zwanenburg A, Leger S, Vallières M, Löck S. Results from the image biomarker standardisation initiative. Radiotherapy and Oncology. 2016;127:S543-S544.
